# Chloroplasts at the Crossroad of Photosynthesis, Pathogen Infection and Plant Defense

**DOI:** 10.3390/ijms19123900

**Published:** 2018-12-05

**Authors:** Yan Lu, Jian Yao

**Affiliations:** Department of Biological Sciences, Western Michigan University, Kalamazoo, MI 49008, USA; jian.yao@wmich.edu

**Keywords:** Photosynthesis, pathogen infection, plant defense, defense-related signaling molecules, chloroplast-targeted effectors, phytotoxins

## Abstract

Photosynthesis, pathogen infection, and plant defense are three important biological processes that have been investigated separately for decades. Photosynthesis generates ATP, NADPH, and carbohydrates. These resources are utilized for the synthesis of many important compounds, such as primary metabolites, defense-related hormones abscisic acid, ethylene, jasmonic acid, and salicylic acid, and antimicrobial compounds. In plants and algae, photosynthesis and key steps in the synthesis of defense-related hormones occur in chloroplasts. In addition, chloroplasts are major generators of reactive oxygen species and nitric oxide, and a site for calcium signaling. These signaling molecules are essential to plant defense as well. All plants grown naturally are attacked by pathogens. Bacterial pathogens enter host tissues through natural openings or wounds. Upon invasion, bacterial pathogens utilize a combination of different virulence factors to suppress host defense and promote pathogenicity. On the other hand, plants have developed elaborate defense mechanisms to protect themselves from pathogen infections. This review summarizes recent discoveries on defensive roles of signaling molecules made by plants (primarily in their chloroplasts), counteracting roles of chloroplast-targeted effectors and phytotoxins elicited by bacterial pathogens, and how all these molecules crosstalk and regulate photosynthesis, pathogen infection, and plant defense, using chloroplasts as a major battlefield.

## 1. Introduction

Photosynthesis, pathogen infection, and plant defense are three important biological processes that have been investigated separately for decades [1]. In algae and plants, photosynthesis occurs in the chloroplast. Photosynthesis could be divided into light reactions and carbon fixation reactions. Photosynthetic light reactions require the participation of four protein complexes in thylakoid membranes (Photosystem II [PSII], cytochrome *b*_6_*f* complex, Photosystem I [PSI], and ATP synthase), and mobile electron carriers plastoquinone, plastocyanin, and ferredoxin. The end products of photosynthetic light reactions are ATP and NADPH; in oxygenic photosynthetic organisms, molecular oxygen (O_2_) is also produced by PSII at this stage, as a water-splitting product. ATP and NADPH produced from photosynthetic light reactions are consumed by photosynthetic carbon fixation in a series of stromal reactions that reduce CO_2_ to triose phosphates. These reactions are catalyzed by ribulose 1,5-bisphosphate carboxylase/oxygenase (Rubisco). The carbohydrates (i.e., triose phosphates) produced by carbon fixation reactions serve as carbon skeletons for the synthesis of primary metabolites such as amino acids [2] and fatty acids [3], phytohormones such as abscisic acid (ABA) [4,5], ethylene (ET) [6], jasmonic acid (JA) [7,8], and salicylic acid (SA) [9,10,11], antimicrobial compounds such as camalexin [12,13], and cell wall reinforcing polymers such as callose and lignin [14]. Synthesis of these primary and specialized metabolites and polymers often requires the consumption of ATP and NADPH, and sometimes, O_2_, the three end products of photosynthetic light reactions. Key steps in the synthesis of defense-related hormones or their precursors occur in the chloroplast. PSII and PSI are also primary generators of singlet oxygen (^1^O_2_) and superoxide (O_2_^⋅−^), respectively [15,16,17]. Reactive oxygen species (ROS) production by the photosynthetic electron transport chain has a protective role over the photosynthetic apparatus when the absorbed excitation energy exceeds the energy consumed during photosynthetic electron transport [15]. On the other hand, excess amounts of ROS damage proteins, lipids, and nucleic acids, and are therefore toxic to many cellular processes, including photosynthesis [15,16,17,18,19,20,21,22,23,24].

All plants grown naturally are attacked by pathogens, such as bacteria, fungi, oomycetes, and nematodes [25]. Plant pathogens have two different lifestyles: necrotrophs and biotrophs [25,26]. Necrotrophs kill plant tissues and gain nutrients from dead tissues; biotrophs keep plant tissues alive and gain nutrients from living cells [25,26]. Hemibiotrophs, such as *Pseudomonas syringae*, a rod-shaped gram-negative bacterium with one to several polar flagella [27], are characterized by an initial biotrophic phase and a later necrotrophic phase [25,26]. Bacterial pathogens enter host tissues through natural openings (e.g., stomata) or wounds [28,29]. Upon invasion, bacterial pathogens utilize a combination of different virulence factors, such as type III effector proteins (T3Es) and phytotoxins, to suppress host defense and promote pathogenicity [30,31,32]. T3Es released by the type III secretion system (T3SS) in gram-negative bacteria are predicted to collectively suppress plant basal defense and reprogram plant photosynthesis and metabolism, to assist pathogen proliferation and nutrition [33,34,35,36,37]. One example of phytotoxins is coronatine (COR), a non-host-specific polyketide produced by many strains of *P. syringae* [30,38]. COR interferes with plant JA signaling by mimicking bioactive JA-isoleucine (JA-Ile) and “fooling” the JA receptor COR-insensitive 1 (COI1) [39,40,41,42]. By modulating plant JA signaling, COR causes stomatal reopening, chlorophyll degradation, and inhibition of SA-mediated defense responses [38].

Plants have developed elaborate constitutive and inducible defense mechanisms to protect themselves from pathogen infections. Constitutive defense includes non-specific antimicrobial toxins and preformed structural barriers (e.g., cell walls) [43]. Inducible defense is triggered by the recognition of pathogen-associated molecular patterns (PAMPs), or effector proteins released by the pathogen [44,45,46]. The recognition of PAMPs by pattern recognition receptors leads to PAMP-triggered immunity (PTI); the recognition of effectors by resistance (R) proteins leads to effector-triggered immunity (ETI) [44,45,46]. Early defense events include cytoskeletal reorganization, cell wall fortification, generation of ROS, stomatal closure, and synthesis of antimicrobial secondary metabolites [21,29,47,48]. Later defense responses include transcription and translation of pathogenesis-related (PR) proteins and the development of the hypersensitive response (a type of programmed cell death [PCD] to minimize pathogen spread) [49,50]. Plants develop the hypersensitive response, a hallmark of ETI, if the pathogen is able to suppress plant basal defense (i.e., the constitutive and inducible defense described above) [44,45,46]. These local defense responses require the participation of multiple defense-related hormones and non-hormone signaling molecules. For example, ABA, JA, SA, ET, ROS, nitric oxide (⋅NO) and Ca^2+^ all function in PAMP-triggered stomatal closure [29]. In addition to local defense at or near the site of infection, plants may develop the systemic acquired resistance (SAR), a “whole-plant” resistance, after a localized exposure to a pathogen [51]. This process is associated with the accumulation of PR proteins and requires the participation of SA [51].

Elaborate interplays exist among photosynthesis, pathogen infection, and plant defense. Here, we review: (1) how defense-related signaling molecules or their precursors are generated in the chloroplast; (2) how these signals crosstalk and regulate photosynthesis and plant defense; (3) how chloroplast-targeted effectors and phytotoxins produced by bacterial pathogens manipulate chloroplastic functions, especially photosynthesis, to suppress plant defense and promote pathogenicity; and (4) why the chloroplast plays a central role in the interplay between photosynthesis, pathogen infection, and plant defense. Plant defense is also regulated by photorespiration and light. If readers are interested in the roles of photorespiration and photoreceptors in plant defense, they may refer to reviews on related topics, such as Kangasjärvi et al. [52] and Ballaré [53].

## 2. The Chloroplast is a Major Synthesis Site for Many Plant Hormones

### 2.1. The Chloroplast is a Major Site of ABA Biosynthesis

ABA is a 15-carbon terpenoid synthesized via 40-carbon carotenoid intermediates, such as zeaxanthin, violaxanthin, and neoxanthin [4,5]. Early steps of ABA biosynthesis, i.e., conversions among different 40-carbon carotenoid intermediates, occur in the chloroplast. It is worth mentioning that the conversion from zeaxanthin to violaxanthin requires the participation of NADPH and O_2_ [54]. 9′-*cis*-neoxanthin and 9′-*cis*-violaxanthin are cleaved into 15-carbon xanthoxin, by 9-*cis*-epoxycarotenoid dioxygenase in the chloroplast. Xanthoxin is then transported to the cytosol and converted into abscisic aldehyde by a short-chain alcohol dehydrogenase. Abscisic aldehyde is oxidized into the final product ABA by an abscisic aldehyde oxidase. This step requires the participation of O_2_ [4,5]. After synthesis, ABA may undergo glycosylation or hydroxylation and become inactive [5,55]. Upon stress, ABA is released from the ABA-glucose conjugate by β-glucosidase [56].

ABA is an important regulator of plant growth and development, biotic stresses, and abiotic stresses [55,57]. ABA induces stomatal closure in response to drought and high salinity [58,59,60]. Stomatal closure limits gas exchange, which is required for photosynthetic carbon fixation reactions [61,62,63]. Consistent with relationships among ABA, stomata, and photosynthesis, exogenous ABA application was found to cause stomatal closure and reduced photosynthesis [61,62,63]. ABA treatment was found to repress the transcription of many plastid genes by both plastid-encoded RNA polymerase and nuclear-encoded plastid RNA polymerase [64]. However, the transcription of *psbD* (*PSII reaction center protein D2*; psb stands for PSII), *psbA* (*PSII reaction center protein D1*), and a few other genes did not respond to ABA treatment [64]. Furthermore, ABA treatment increased the transcription of four nuclear-encoded genes: *RSH2* (*RelA/SpoT homolog 2*), *RSH3* (*RelA/SpoT homolog 3*), *PTF1* (*plastid transcription factor 1*), and *SIG5* (*sigma factor 5*) [64]. RSH2 and RSH3 catalyze the synthesis of guanosine-3′-5′-bisdiphosphate (ppGpp) [65], an inhibitor of the plastid-encoded plastid RNA polymerase (PEP) [66]. Therefore, ABA may inhibit gene expression in the chloroplast by stimulating ppGpp synthesis [64]. PTF1 [67] and SIG5 [68] are chloroplast-targeted transcription factors required for the transcription of *psbD*. ABA may activate the transcription of *psbD* by promoting transcription initiation at the blue light responsive promoter, which requires the participation of PTF1 and SIG5 [64].

The roles of ABA in plant defense against pathogens are multifaceted. ABA induces stomatal closure in response to pathogen attacks; therefore, ABA is important in blocking the entry of bacterial pathogens via stomata [28,29]. The core components of ABA-mediated stomatal immunity include the regulatory component of ABA receptor (RCAR), 2C-type protein phosphatase (PP2C), and serine/threonine (Ser/Thr) protein kinase OST1 (open stomata 1) [69]. ABA-mediated stomatal closure involves other signaling molecules. For example, ABA application induced ROS production in guard cells, mediated by ABA-activated OST1 [70,71]. OST1 promotes hydrogen peroxide (H_2_O_2_) production by phosphorylating the respiratory burst oxidase homologue NADPH oxidase F (RbohF) located on the guard cell plasma membrane [72]. ABA treatment also resulted in increased *Rboh1* (a tomato homolog of *Arabidopsis thaliana RbohF*) gene expression, increased NADPH oxidase activity, and increased apoplastic and chloroplastic H_2_O_2_ concentrations, at the whole seedling scale [73]. It should be noted that, although ABA promotes ROS production in non-seed tissues, this hormone suppresses ROS production in imbibed seeds [74,75]. ABA treatment induced ⋅NO production in guard cells, mediated by nitrate reductase and a ⋅NO synthase-like enzyme [76,77,78,79]. Furthermore, ABA treatment caused sustainable increases of calcium ions (Ca^2+^) in *Commelina communis* and *Arabidopsis* guard cells [80]. The sequential H_2_O_2_, ⋅NO, and Ca^2+^ spikes are required for ABA-induced reduction of guard cell turgor and subsequent stomatal closure as well as ABA-induced gene expression in the guard cell nucleus [80,81].

ABA suppresses the post-invasion PTI basal response [82]. ABA-hypersensitive *Arabidopsis* mutants displayed increased susceptibility to *P. syringae* while *Arabidopsis* mutants with defective ABA synthesis or perception showed increased resistance [83,84]. In addition, exogenous ABA application results in increased susceptibility of many plant species to bacterial and fungal pathogens [83,85,86,87,88,89,90]. For example, ABA pretreatment caused reduced callose deposition and enhanced multiplication of *P. syringae* pv. *tomato* (*Pst*) wild-type strain DC3000 in *Arabidopsis* [83].

**Figure 1:**
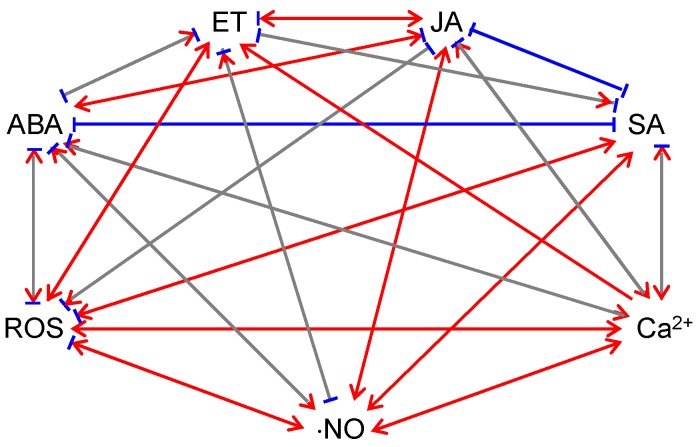
Interactions among defense-related signals. Red arrow heads represent positive (promoting) effects; blue bars represent negative (inhibitory) effects; grey lines with red arrow heads and blue bars represent both positive and negative effects. This is not an exhaustive presentation of all defense-related signals, but it shows the major ones discussed in this review.

ABA influences the production and signaling of other hormones (Figure 1). The JA signaling pathway contains two distinct and antagonistic branches: the ERF (ethylene response factor)-branch is responsible for activating pathogen-responsive genes and repressing wounding-responsive genes and is co-regulated by ET; the MYC (myelocytomatosis) branch is responsible for activating wounding-responsive genes and repressing pathogen-responsive genes, which is co-regulated by ABA [91,92]. *Arabidopsis* plants constitutively expressing MYC2, a key transcriptional activator of JA responses, were hypersensitive to both JA and ABA [93]. Several studies showed that ABA has synergistic impacts on the MYC branch and antagonistic impacts on the ERF branch [93,94,95]. ABA has negative effects on SA signaling. ABA was found to locally down-regulate SA biosynthesis by transcriptional regulation of isochorismate synthase 1 (ICS1, i.e., SA-induction-deficient 2 [SID2]), an SA biosynthetic enzyme, which resulted in suppression of SA-induced defenses [96]. ABA treatment also suppressed SAR development via inhibiting SA signaling and this process appeared to be independent of JA/ET signaling [97]. Both endogenous ABA levels and pathogen effector-induced increases of ABA are involved in the antagonism between ABA and SA [96]. The effector-mediated manipulation of ABA biosynthesis and signaling is a key virulence mechanism for pathogens [83]. JA and SA signaling has reciprocal antagonistic interactions [98,99,100]. Therefore, ABA may indirectly antagonize SA signaling via its activation effect on JA signaling [101].

### 2.2. Methionine, the Precursor for ET Biosynthesis, is Made in the Chloroplast

ET is a two-carbon gaseous plant hormone. ET biosynthesis is a three-step process: (1) the conversion of methionine (Met) to S-adenosyl Met (SAM) by SAM synthetase; (2) the conversion of SAM to 1-aminocyclopropane-1-carboxylic acid (ACC) by ACC synthase; and (3) the conversion of ACC to ET by ACC oxidase [6]. Although ET itself is not produced in the chloroplast, Met, the precursor of ET biosynthesis, is made in the chloroplast. There are three cobalamin-independent Met synthases (MSs) in *Arabidopsis* [102]. MS3 is located in the chloroplast and is required for Met generation from homocysteine synthesized *de novo* in chloroplasts [102]. MS1 and MS2 are present in the cytosol and are most likely involved in Met regeneration from homocysteine made during the activated methyl cycle [102].

ET is another important regulator of plant growth and development, abiotic stresses, and biotic stresses [6,103]. ET may influence photosynthesis by regulating stomatal aperture, although the effects of ET on stomatal movements varied in the literature. ET was reported to modulate stomatal opening in different species when epidermal peels were used [104,105,106]. However, ET was found to mediate stomatal closure when intact leaves were used [107,108,109]. ET may impact photosynthesis by regulating chlorophyll contents, *chlorophyll a/b-binding protein* (*CAB*) gene expression, PSI and PSII efficiency, and Rubisco activity, on an age-dependent manner [110]. ET-insensitive *Arabidopsis* and tobacco mutants displayed reduced chlorophyll contents, lower Rubisco activity, and decreased *CAB* expression in juvenile non-senescing leaves [111,112,113,114], suggesting that the basal-level ET perception is required for normal photosynthetic capacity in juvenile non-senescing *Arabidopsis* and tobacco leaves [110]. However, the opposite is true in mature senescing leaves of ET-insensitive *Arabidopsis*, tomato, and tobacco mutants [111,115,116,117]. These age-dependent responses suggest that ET is needed for normal chlorophyll accumulation in young non-senescing leaves but may promote chlorophyll degradation in mature senescing leaves [110]. It should be noted that ET application on juvenile non-senescing *Arabidopsis* leaves resulted in reduced chlorophyll levels and reduced *CAB* transcript contents [111,118], indicating that excess amounts of ET inhibits photosynthesis [110]. The influence of ET on photosynthesis is also species-specific [110]. The ACS-deficient maize mutant displayed increased levels of chlorophyll and Rubisco and improved leaf performance [119]. Furthermore, exogenous ET treatment did not affect photosynthesis in maize plants [120,121,122].

The roles of ET in plant defense against pathogens are also multifaceted. As described above, ET modulates stomatal closure in intact leaves; therefore, ET is important in blocking the entry of bacterial pathogens via stomata. ET-mediated stomatal closure involves other signaling molecules. For example, ET-induced stomatal closure was found to be dependent on apoplastic H_2_O_2_ production by RbohF NADPH oxidase located on the guard cell plasma membrane [109], peroxidase on the guard cell wall [123], and polyamine oxidase in the guard cell nucleus, cytoplasm, and cell wall [124]. Intriguingly, ET was found to inhibit ABA-induced stomatal closure by reducing the ⋅NO content in *Vicia faba* guard cells [79].

ET plays different roles in plant defense, depending on pathogen types [6]. In general, ET obstructs symptom development caused by necrotrophic pathogens and promotes cell death caused by biotrophic and hemibiotrophic pathogens [125,126,127]. For example, *Arabidopsis* mutants with decreased ET sensitivity demonstrated enhanced susceptibility to the necrotrophic fungus *Botrytis cinerea* but enhanced resistance to the hemibiotrophic pathogen *Pst* and the biotrophic pathogen *Xanthomonas campestris* pv. *campestris* [125,126]. Likewise, ET-insensitive soybean mutants showed more severe symptoms when infected with necrotrophic fungi *Septoria glycines* and *Rhizoctonia solani*, but displayed less severe symptoms when infected with hemibiotrophs *P. syringae* pv. *glycinea* and *Phytophthora sojae* [127].

ET influences the production and signaling of other hormones (Figure 1). ET has negative effects on ABA signaling. As described previously, ABA application induced ⋅NO production and subsequent stomatal closure [76,77,78,79]; however, ET treatment reversed the effects of ABA on ⋅NO production and stomatal closure [79]. The negative effects of ET on ABA signaling were also observed in root growth and seed germination [128,129,130]. ET has positive effects on JA signaling [92,131]. ET synergizes the ERF branch of the JA-signaling pathway to activate the expression of genes involved in defense against necrotrophic pathogens [93,132,133]. Depending on the pathosystem, ET may have positive or negative impacts on SA signaling [92,134]. For example, EIN3 (ethylene insensitive 3) and EIL1 (ethylene insensitive 3-like 1), two transcription factors involved in ET signaling, were found to repress the expression of ICS1 [135], the isochorismate synthase required for pathogen-induced SA biosynthesis. Loss-of-function *ein3* and *eil1 Arabidopsis* mutants displayed enhanced resistance to *P. syringae*; on the contrary, *Arabidopsis* plants overexpressing EIN3 exhibited enhanced susceptibility to *P. syringae* [135]. Furthermore, the *ein3-1 eil1-1* double mutant over-accumulated SA in the absence of pathogen infections [135]. This study demonstrated that in the *Arabidopsis-P. syringae* pathosystem, ET acts negatively on SA-mediated defense [135]. However, in oilseed rape, ET acted positively on SA-mediated resistance against *Leptosphaeria maculans*, a fungal pathogen [136].

### 2.3. The Chloroplast is also a Site of JA Synthesis

JA is a 12-carbon oxygenated fatty acid derivative, subsequently synthesized in chloroplasts and peroxisomes [7,8]. In the chloroplast, the 18-carbon fatty acid linolenic acid (18:3) is released from membrane lipids and then oxidized at the C-13 position by a lipoxygenase. This reaction requires O_2_ as a substrate. The oxidized intermediate is cyclized into 12-oxo-phytodienoic acid (OPDA) by the combined action of allene oxide synthase and allene oxide cyclase. After being transported to the peroxisome, OPDA is reduced into 3-oxo-2-(2-pentenyl)-cyclopentane-1-octanoic acid (OPC-8) by OPDA reductase. This reaction consumes NADPH. OPC-8 is then converted to JA via β-oxidation, which requires the participation of ATP and O_2_. Once synthesized, JA may undergo amino acid conjugation, methylation, sulfonation, glucosylation, and hydroxylation [8]. These chemical modifications allow fine-tuning of the accumulation, activity, and mobility of JA [8].

JA also is a regulator of plant growth and development, abiotic stresses, and biotic stresses [7,8]. Exogenous treatment of potato leaves with 1 μM JA caused significant reduction in the amounts of photosynthetic pigments [137]. Furthermore, methyl JA-treated *Arabidopsis* protoplasts as well as *Vicia faba* and rice seedlings displayed compromised photosynthetic electron transport and carbon fixation reactions [138]. These photosynthetic defects were attributed to ROS accumulation as pre-incubation of samples with an antioxidant or ROS scavenger offered significant protection [138].

JA is involved in defense against necrotrophic pathogens and herbivorous insects [7,8]. JA and ET act synergistically upon attack by necrotrophs; JA and ABA act synergistically during herbivory [7,8]. Necrotrophic pathogen attacks induce the expression of JA- and ET-responsive genes and defense against these pathogens [7,8]. Methyl JA treatment was found to up-regulate the expression of genes in JA biosynthesis, defense responses, oxidative stress responses, senescence, and cell wall modification, and down-regulate the expression of genes in chlorophyll biosynthesis and photosynthesis [139]. This general trend was also observed at the protein level [140]. JA is also a central player in induced systematic resistance (ISR), which is induced by nonpathogenic microbes, such as plant growth-promoting rhizobacteria and mycorrhiza, upon root colonization [134]. ISR confers a broad spectrum of resistance to future pathogen invasion in many plant species [134]. ISR was blocked in *Arabidopsis* JA-signaling mutants [141,142,143], demonstrating the role of JA in ISR.

JA influences the production and signaling of other hormones (Figure 1). JA may exert positive impacts on ABA signaling under abiotic stress. For example, JA application caused increased ABA levels in barley and citrus seedlings [144]. JA accumulation appeared to be needed for ABA build-up under drought conditions [145,146], consistent with the notion that ABA and JA act synergistically under drought [147]. As mentioned previously, JA and ABA act synergistically on the MYC branch of JA signaling to activate wounding-responsive genes and repress pathogen-responsive genes while JA and ET act synergistically on the ERF branch to activate pathogen-responsive genes and repress wounding-responsive genes [91,92]. Furthermore, depending on the plant species and growth stage, methyl JA application may promote or inhibit ET production in seedlings, fruits, and seeds [148].

JA influences the production and signal transduction of non-hormone signaling molecules (Figure 1). For example, methyl JA treatment caused sequential increases of mitochondrial and chloroplastic H_2_O_2_ in *Arabidopsis* protoplasts, which led to mitochondrial aggregation and swelling, photosynthetic dysfunction, chloroplast morphology changes, and ultimately, cell death [138]. Methyl JA-induced H_2_O_2_ accumulation was also observed in guard cells and intact seedlings [138,149]. However, Methyl JA was also found to inhibit cell wall damage-triggered ROS accumulation in *Arabidopsis* seedlings [150]. JA application enhanced ⋅NO production in *Vicia faba* guard cells [151]. Furthermore, JA treatment induced the increase of cytosolic cAMP, which led to activation of cyclic nucleotide-gated channel 2 (CNGC2) on the plasma membrane and apoplastic Ca^2+^ influx via CNGC2 [152].

### 2.4. The Chloroplast is Involved in the Synthesis of SA

SA is a 7-carbon phenolic acid synthesized via two routes: the isochorismate pathway in the chloroplast and the phenylalanine (Phe) ammonia-lyase pathway. In the chloroplast, chorismate is isomerized to isochorismate by isochorismate synthase (ICS); isochorismate is then converted to SA and pyruvate by isochorismate pyruvate lyase. Gene(s) encoding isochorismate pyruvate lyase in plants have not been identified yet. In *Arabidopsis*, there are two *ICS* genes: *ICS1* and *ICS2* [153,154]. Under pathogen infection or stress conditions, the *ics1* mutants accumulated ~5–10% and the *ics1 ics2* double mutant accumulated ~4% of wild-type levels of SA [153,154]. These genetic studies demonstrated that the isochorismate pathway is the primary route of SA biosynthesis in plants [153,154]. In the chloroplast, chorismate is also converted to Phe, which is exported to the cytosol [9,10,11]. In the cytosol, Phe is converted to cinnamate by Phe ammonia-lyase; cinnamate is then converted to SA via β-oxidation or non-oxidative routes. After synthesis, SA may be modified via glucosylation, methylation, amino acid conjugation, sulfonation, and hydroxylation, which allows fine regulation of its accumulation, activity, and mobility [10,11].

SA plays regulatory roles in plant growth and development, abiotic stresses, and biotic stresses [10,11]. The effects of SA treatment on photosynthesis under optimal growth conditions are controversial [155]. Spraying soybean shoots with SA solutions significantly improved plant growth although the photosynthetic rate was not affected [156]. Other researchers reported that foliar application of SA at an optimal concentration (10^−5^ μM) on Indian mustard, maize, and soybean plants resulted in significant increases in photosynthetic rates while SA treatment at higher concentrations had inhibitory effects on photosynthesis [157,158,159]. Under stress conditions, pretreatment with SA minimized the detrimental effects of stress factors and thus resulted in higher photosynthetic capacity [160,161,162]. Taken together, the effects of SA application on photosynthesis are dependent on plant species, application methods and durations, and growth conditions [155]. The SA-feeding experiments also suggest that delicately regulated SA levels are needed for optimum photosynthesis [155]. A similar conclusion was drawn from physiological characterization of *Arabidopsis* mutants with constitutively high or low SA contents [163]. Under standard growth conditions, all the mutants displayed suboptimal photosynthesis and a dwarf phenotype [163].

SA and its derivate methyl SA induce local resistance at the site of infection and SAR at the whole-plant level [10,11]. Examples of SA-induced local resistance include localized cell death and defense gene expression [10,11]. To develop SAR, a signal from the infected leaf is transmitted to other parts of the plant via the phloem. Recent studies showed that methyl SA acts as a phloem-mobile signal [164]. When the accumulation of methyl SA at primary infected tobacco leaves was suppressed by silencing SA methyl transferase, SAR was compromised [164]. In *Arabidopsis*, SA-induced SAR is primarily mediated by NPR1 (nonexpressor of PR genes 1) [165,166,167]. In uninfected plants, NPR1 exists as disulfide-linked oligomers in the cytoplasm [168]. Upon pathogen infection, the increase in SA results in reduction of disulfide bonds in NPR1, monomerization, and subsequent translocation to the nucleus, where NPR1 activates the expression of *PR* genes [168]. S-nitrosylation of cysteine (Cys) 156 in NPR1 facilitates its oligomerization and retention in the cytoplasm, whereas disulfide reduction by SA-activated thioredoxin promotes monomerization and nuclear translocation [168]. In rice, SA signaling is branched into the NPR1-dependent and the WRKY45 transcription factor-dependent pathways [169]. Although SA and JA are both classified as defense hormones, they use different strategies against pathogens. While JA protects plants against necrotrophs and insect herbivores, SA is predominantly involved in plant defense against biotrophs and hemibiotrophs [10,99,100].

SA influences the production and signal transduction of other hormones (Figure 1). The activation of SAR by SA directly suppresses the expression of ABA biosynthetic and responsive genes [97]. This suppression is likely mediated by NPR1 or signals downstream of NPR1 [97]. In line with this hypothesis, NPR1 overexpression in rice significantly negated the enhancement of blast susceptibility by ABA [90]. As a hormone for defense against biotrophs, SA suppresses JA-ET defense responses and thus increases plant susceptibility against necrotrophs [10,99,100]. For example, SA and aspirin (the acetylated form of SA) were found to suppress ET biosynthesis and expression of JA/ET-inducible wounding-responsive genes [166,170,171,172,173]. Recent studies showed that SA suppresses JA-ET signaling downstream of the E3 ubiquitin ligase Skip-Cullin-F-box complex SCF^COI1^ [174,175,176]. Ubiquitination is a strategy used by both the plant and the pathogen [177,178,179]. From the plant’s point of view, ubiquitination-triggered degradation of pathogen proteins (e.g., effectors) is protective [177,178,179]. On the other hand, pathogens developed mechanisms to utilize or evade ubiquitination, to manipulate plant responses [177,178,179].

SA influences the production and signal transduction of non-hormone signaling molecules (Figure 1). A number of studies showed that SA application promotes ROS accumulation [180,181,182]. Consistent with this observation, genetic and pharmacological analyses demonstrated that high and low SA contents were strictly correlated with high and low foliar H_2_O_2_ concentrations, respectively [163,183]. How does SA induce ROS accumulation? SA was found to inhibit ROS scavenging enzymes, such as catalase and ascorbate peroxidases [180,182,183,184]. Interestingly, evidence also showed that SA may promote ROS scavenging under stress conditions [163,185,186]. This is accomplished by inducing the accumulation of glutathione and reducing power [163,187]. Taken together, SA has an ambivalent effect on ROS accumulation and scavenging [188], depending on treatments and growth conditions. SA promoted ⋅NO production by a ⋅NO synthase-like enzyme in *Arabidopsis* on a dose-dependent manner [189]. SA treatment induced the increase in cytosolic Ca^2+^ concentration in tobacco suspension culture cells [190,191].

## 3. The Chloroplast is a Major Site of Free Radical Production

### 3.1. The Photosynthetic Electron Transport Chain is a Major Site of ROS Production

ROS are reactive molecules and free radicals derived from O_2_. Examples of ROS include O_2_^⋅−^, H_2_O_2_, ^⋅^OH (hydroxyl radical), and ^1^O_2_. Different ROS have different half-lives and specificities [192]. ROS can be produced in the apoplast, chloroplasts, mitochondria, and peroxisomes of a plant cell [21,192,193]. Increases in ROS accumulation have been detected in both PTI and ETI [21]. The biphasic accumulation of ROS in different subcellular compartments is the hallmark of successful recognition of pathogens by plants [193]. The first, low-amplitude, and transitory phase occurs within minutes after infection and is mostly apoplastic and tightly linked to the activities of plasma-membrane Rboh NADPH oxidases (e.g., RbohD and RbohF) and cell-wall peroxidases [21,193,194]. O_2_^⋅−^ produced by NADPH oxidases is rapidly converted to H_2_O_2_ either spontaneously or by superoxide dismutase (SOD) [193]. The resulting H_2_O_2_, along with H_2_O_2_ generated by cell-wall peroxidases, crosses the plasma membrane and enters the cell via free diffusion or aquaporin-facilitated diffusion [195,196,197]. The second, high-amplitude, and sustained phase takes place a few hours after infection and is typically associated with the establishment of defense responses and the hypersensitive response [21,193,194]. The second phase happens in multiple compartments, including the apoplast, chloroplasts, mitochondria, and peroxisomes [193].

In the chloroplast, PSI and PSII are two main sources of ROS production [15,16]. PSI is a primary generator of O_2_^⋅−^ [198,199]. During photosynthesis, O_2_ is continuously reduced to O_2_^⋅−^ by PSI, and O_2_^⋅−^ is quickly converted to H_2_O_2_ and O_2_ by the Cu-Zn-SOD attached to PSI [15,16]. PSII is a major generator of ^1^O_2_ [200,201]. During photosynthesis, ground-state oxygen (^3^O_2_) is continuously excited to ^1^O_2_ by triplet-excited-state chlorophyll (^3^P680*) in the PSII reaction center. ROS production has both positive and negative effects on photosynthesis. On the one hand, ROS production by the two photosystems acts as alternative electron sinks, alleviating the negative impacts of over-reduction and photo-inactivation of the photosynthetic apparatus [15,202]. In this regard, chloroplastic ROS production protects the photosynthetic apparatus, especially when the absorbed excitation energy exceeds the energy consumed by the photosynthetic electron transport chain [15,16]. On the other hand, non-physiological concentrations of chloroplastic ROS may cause irreversible damage to thylakoid membranes and photosynthetic components, by lipid peroxidation, protein damage, membrane destruction, and ion leakage [203,204].

During plant defense against pathogens, ROS have a variety of functions, including (1) killing pathogens directly [205], (2) strengthening cell walls [206], (3) activating defense gene expression [165,207], (4) mediating lipid peroxidation (to execute localized cell death) [208], (5) causing phytoalexin accumulation (to inhibit pathogen growth) [209], (6) inducing the hypersensitive response [210], (7) modulating vesicle trafficking (to mediate signaling) [211], and (8) being required for the internalization of pattern recognition receptors [212]. Similar to ROS produced in other cellular compartments, chloroplastic ROS are essential for the hypersensitive response in plants [213,214,215,216]. For example, infiltration of wild-type tobacco leaves with a non-host pathogen *Xanthomonas campestris* pv. *vesicatoria* (*Xcv*) resulted in increased ROS accumulation, preceding the appearance of localized cell death [215]. However, in *Xcv*-inoculated tobacco plants with compromised chloroplastic ROS production, localized cell death was significantly reduced [215]. These tobacco plants expressed cyanobacterial flavodoxin, which prevents chloroplastic ROS production during pathogen infection [215]. The chloroplast has a number of enzymatic ROS scavenging systems, such as SOD, ascorbate peroxidase (APX), glutathione peroxidase (GPx), and the thioredoxin-peroxiredoxin (TRX-PRX) system [15,16,217]. Overexpression of thylakoid-bound APX resulted in delayed hypersensitive response and reduced symptoms [213]. On the contrary, silencing of *PRX* genes resulted in enhanced spreading of *Pst* wild-type strain DC3000-induced PCD and enhanced bacterial growth and disease susceptibility [216]. These symptoms were absent when the plants were inoculated with a COR-deficient *Pst* strain DB29 [216]. Taken together, these results suggest that chloroplastic ROS scavenging is critical to a plant’s hypersensitive response and that COR-producing bacterial strains may influence the homeostasis of chloroplastic ROS in a COR-dependent manner [216]. Chloroplastic ROS also up-regulate the expression of defense-related genes in the nucleus. By silencing thylakoid-bound APX, it was discovered that over-accumulation of chloroplastic H_2_O_2_ resulted in up-regulation of nuclear genes involved in pathogen defense [218]. The retrograde transcriptional reprogramming induced by chloroplast-generated ROS could potentially be achieved by (1) diffusion of ROS from the chloroplast to other subcellular compartments (e.g., the cytosol and then the nucleus) [219,220], (2) manipulating hormone (e.g., JA and SA) signaling [218], and (3) influencing the integrity of the chloroplast envelope [221,222,223].

ROS influences the production and signaling of phytohormones (Figure 1). H_2_O_2_ acts as a secondary messenger in ABA signaling and mediates ABA-induced stomatal closure [224,225]. Both H_2_O_2_ and ABA treatments activated mitogen-activated protein kinase (MAPK) and up-regulated the expression and activities of antioxidant enzymes [226,227,228,229]. Pretreating plants with ROS inhibitors (diphenylene iodonium and imidazole [NADPH oxidase inhibitors]) or scavengers (Tiron [O_2_^⋅−^ scavenger] and dimethylthiourea [H_2_O_2_ scavenger]) blocked such enhancements [230]. These observations suggest that ROS are required in ABA-induced antioxidant defense [226,227,228,229,230]. Interestingly, H_2_O_2_ was found to up-regulate ABA catabolic genes during *Arabidopsis* seed imbibition, which resulted in a lower ABA level [231]. The antagonism between ROS and ABA in seed germination was also observed in monocots [74,75]. ROS play a positive role in ET signaling. For instance, H_2_O_2_ induced the transcription of ET synthetic genes in etiolated *Brassica oleracea* seedlings [232]. O_2_^⋅−^ generators promoted ET synthesis in etiolated mung bean seedlings [233]. Furthermore, ET-induced stomatal closure was dependent on H_2_O_2_ synthesis in *Arabidopsis* guard cells [109]. ROS have a negative impact on JA signaling. ROS negated cell wall damage-triggered JA production in *Arabidopsis* seedlings [150]. Over-accumulation of chloroplastic H_2_O_2_ up-regulated the expression of transcription factors that negatively regulate JA signaling (e.g., jasmonate-zim-domain [JAZ] proteins) [234]. ROS play a positive role in SA signaling. H_2_O_2_ over-accumulation in the chloroplast resulted in elevated SA levels and enhanced SA responses [218]. It was proposed that the elevated SA level assists the accumulation of H_2_O_2_, to control the propagation of cell death [235].

ROS influences the production and signal transduction of other non-hormone signaling molecules (Figure 1). The development of the hypersensitive cell death requires balanced synthesis of ROS and ⋅NO, as well as physical interaction between H_2_O_2_ and ⋅NO [236]. In addition, ABA-induced ⋅NO production was dependent on ABA-induced H_2_O_2_ production in *Arabidopsis* guard cells [237]. Furthermore, H_2_O_2_ treatment caused a transient cytosolic Ca^2+^ burst in tobacco suspension cells [238]. Ca^2+^ burst in the plant cell is an important early event during plant defense. This phenomenon was also observed in guard cells and other cell types of additional plant species [239,240,241].

### 3.2. The Chloroplast is a ⋅NO Generator

⋅NO is a gaseous free radical. ⋅NO is primarily produced in the apoplast, chloroplasts, mitochondria, and peroxisomes of a plant cell [242]. There are two independent ⋅NO production pathways in the chloroplast: (1) reductive ⋅NO generation from nitrite catalyzed by the thylakoid membrane-associated nitrate reductase; and (2) oxidative ⋅NO synthesis from arginine (Arg) catalyzed by a ⋅NO synthase-like enzyme [243,244]. Nitrate reductase generally reduces nitrate to nitrite, but this enzyme can also reduce nitrite to ⋅NO [245,246]. It is worth mentioning that the production of ⋅NO by nitrate reductase and the ⋅NO synthase-like enzyme requires NAD(P)H [246].

⋅NO modulates various aspects of plant growth, development, and stress responses [242,247,248]. For example, exogenous ⋅NO has been shown to inhibit photosynthesis in intact leaves and the ⋅NO concentration causing inhibition to net photosynthesis is much lower than those required for visible injury [249,250]. ⋅NO may regulate the activities of target proteins via metal center-binding, tyrosine (Tyr) nitration, and Cys S-nitrosylation [248]. ⋅NO inhibits PSII electron transfer by binding reversibly to three sites in PSII [251]: the non-heme iron between Q_A_ and Q_B_ [252,253], the Mn cluster of the oxygen-evolving complex [254], and the redox-active Tyr residue in D2 [255]. The first two sites involve interaction with metal centers while the third site involves Tyr nitration [252,255,256]. ⋅NO could also inhibit photosynthetic carbon fixation (i.e., Rubisco activity) via S-nitrosylation of Cys^65^ in the Rubisco small subunit [257].

⋅NO plays a variety of roles in plant defense against pathogens. As mentioned previously, a concerted production of ROS and ⋅NO and the interaction between H_2_O_2_ and ⋅NO are required for the development of the hypersensitive cell death [236]. On the one hand, ⋅NO potentiates ROS-induced localized cell death and induces defense gene expression [258]. On the other hand, as the concentration of S-nitrosothiols increases during the oxidative and nitrosative bursts, ⋅NO induces S-nitrosylation of the RbohD NADPH oxidase at Cys^890^, limiting further ROS production and cell death [259]. In addition, ⋅NO may enable local resistance and SAR, by inducing SA accumulation [260]. Furthermore, ⋅NO may induce the expression of defense-related genes via Ca^2+^-dependent (or –independent) pathways [261].

⋅NO influences the production and signaling of phytohormones (Figure 1). ⋅NO participates in the regulation of stomatal movements: ABA and ⋅NO both induce stomatal closure; ABA promotes ⋅NO production [76,78,262]. Conceivably, ⋅NO scavenging and impaired ⋅NO generation inhibit ABA-induced stomatal closure [76,78,262]. However, ⋅NO was recently found to inhibit the activity of OST1 (a positive regulator of H_2_O_2_ production) in guard cells via S-nitrosylation, suggesting that ⋅NO may also act as a negative regulator of ABA signaling [263]. ⋅NO counteracts ABA in seed dormancy release and germination as well [264]. For example, ⋅NO is involved in H_2_O_2_-mediated up-regulation of ABA catabolism during seed imbibition [231]. ⋅NO is necessary for biphasic ET generation during the hypersensitive response [265,266]. The positive effect of ⋅NO on ET production is also seen under hypoxia, during which over-accumulation of ⋅NO triggers ET synthesis, possibly via S-nitrosylation of ET biosynthetic enzymes, e.g., ACC synthase and ACC oxidase [267]. ⋅NO could also have negative effects on ET production. For example, ⋅NO may inhibit ET biosynthesis via S-nitrosylation of SAM synthase [268] or by forming an inhibitory complex with ACC oxidase [269]. ⋅NO contributes positively to JA production, by initiating the expression of JA synthetic genes [270,271,272]. ⋅NO may have positive effects on SA generation. Treating tobacco leaves with ⋅NO synthase resulted in increased SA levels and initiation of both SA-dependent and SA-independent gene expression [270,273]. ⋅NO plays a paradoxical role in SA-mediated SAR. Although ⋅NO induces SA accumulation, promoting NPR1 monomerization and translocation from the cytoplasm to the nucleus, ⋅NO also initiates S-nitrosylation of NPR1, keeping NPR1 in the oligomeric form in the cytoplasm [168].

⋅NO influences the production and signal transduction of other non-hormone signaling molecules (Figure 1). ⋅NO could physically interact with ROS and form reactive nitrogen species (RNS), e.g., peroxynitrite (ONOO^−^), nitrogen dioxide (⋅NO_2_), and dinitrogen trioxide (N_2_O_3_) [274]. As described above, ⋅NO have positive and negative effects on ROS signaling [258]. At the early stage of pathogen infection, ⋅NO potentiates the induction of the hypersensitive cell death by ROS [258]; at the later stage, ⋅NO reduces additional ROS production and cell death by promoting S-nitrosylation of RbohD NADPH oxidase [259]. In addition, ⋅NO and ROS act in concert with ABA to regulate stomatal movements [237]. Furthermore, ⋅NO may activate intramolecular Ca^2+^ channels and induce cytosolic Ca^2+^ spikes [77].

## 4. The Chloroplast is a Site for Ca^2+^ Signaling

Extracellular stimuli induce Ca^2+^ spikes in the cytoplasm as well as other organelles such as chloroplasts [275]. PAMP (e.g., flagellin and chitin) treatment was found to induce a rapid Ca^2+^ spike in the cytosol, followed by a long-lasting Ca^2+^ spike in the chloroplast stroma [276]. The stromal Ca^2+^ spike was substantially reduced in *cas-1*, an *Arabidopsis* mutant lacking a thylakoid-membrane-localized Ca^2+^-sensing protein (CAS) [276]. However, the cytosolic Ca^2+^ spike was not impaired in the *cas-1* mutant [276]. Therefore, it was proposed that CAS is involved in the generation of stromal Ca^2+^ spikes by releasing Ca^2+^ from thylakoid membranes [276].

As an essential mineral element, Ca^2+^ is required in cell wall synthesis, cell division, and membrane functions. It was reported that a low level of Ca^2+^ is required for normal photosynthesis in sugar beets [277]. CAS and stromal Ca^2+^ spikes participate in the regulation of photosynthesis in response to abiotic stresses. Using *Chlamydomonas reinhardtii* CAS knockdown lines (*cas-kd*), it was demonstrated that CAS is required for the high light tolerance of photosynthetic light reactions [278]. When transferred under high light, the *cas-kd* lines could not induce the expression of LHCSR3 (light-harvesting complex stress-related 3), a protein essential for non-photochemical quenching [278]. Under prolonged high light exposure, the *cas-kd* lines displayed severe light sensitivity and the activity and recovery of PSII were almost abolished [278]. These defects could be fully rescued by a 10-fold increase in the Ca^2+^ concentration in the growth medium [278]. Consistent with these observations, foliar Ca^2+^ pretreatment was found to alleviate the adverse effects of stress factors and improve photosynthesis in many plant species [279,280,281,282]. Taken together, these results showed that stromal Ca^2+^ spikes and CAS are critical for the regulation and photoacclimation of photosynthesis.

CAS and stromal Ca^2+^ spikes are involved in both PTI and ETI. The *cas-1* mutant demonstrated severely impaired resistance to virulent and avirulent *Pst* strains [276]. PTI responses (e.g., stomatal closure, callose deposition, and accumulation of defense-related compounds) were substantially compromised in the *cas-1* mutant [276]. ETI responses, such as localized cell death, were also delayed and suppressed in the *cas-1* mutant [276]. Biochemical characterization of *CAS*-silenced *Nicotiana benthamiana* plants suggested that CAS probably functions downstream of the MAPK signaling cascade and upstream of ROS signaling and SA accumulation [276].

Ca^2+^ influences the production and signal transduction of phytohormones (Figure 1). Increases in cytosolic Ca^2+^ are a common feature of ABA-mediated stomatal movements and ABA-regulated nuclear gene expression [80]. Ca^2+^ is also required in ET-mediated pathogen responses [283]. When Ca^2+^ fluxes are blocked by chelators, ET-dependent induction of chitinase accumulation was inhibited, but ET-independent induction was not affected [283]. Interestingly, exogenous Ca^2+^ application was found to decrease ABA accumulation but increase ET production in *Fusarium culmorum* (a fungal pathogen)-treated wheat seedlings [284]. Therefore, it was proposed that Ca^2+^ influences ET-ABA balance in plants [284]. Using the Ca^2+^ channel blocker heparin, it was shown that changes in the cytosolic Ca^2+^ concentration are important in JA signaling [285]. Heparin treatment also promoted the expression of JA-responsive genes, indicating that the release of Ca^2+^ from intracellular stores suppresses the expression of JA-responsive genes [285]. Ca^2+^ also regulates SA-mediated plant immunity and the regulation is mediated through calmodulin, a Ca^2+^/calmodulin-binding transcription factor SR1 (signal responsive protein 1), and a positive regulator of SA level – EDS1 (enhanced disease susceptibility 1) [286]. As a negative regulator of plant immunity, SR1 binds to the promoter region of EDS1 and inhibits EDS1 expression [286]. The binding of Ca^2+^/calmodulin to SR1 is required for the suppression role of SR1 [286].

Ca^2+^ influences the production and signal transduction of other non-hormone signaling molecules (Figure 1). Ca^2+^ has a positive impact on ROS production. When Ca^2+^ signaling is blocked by chelators or channel blockers, elicitation of the oxidative burst was prevented [150,287]. Ca^2+^ also regulates ROS production via protein kinases, which phosphorylate RbohB NADPH oxidase in a Ca^2+^-dependent manner [288,289]. Ca^2+^ is also essential to ⋅NO production as plant ⋅NO synthase-like enzymes require Ca^2+^ and calmodulin as cofactors [258,290,291].

## 5. The Complex Relationship between Photosynthesis and Defense-related Signals

**Figure 2:**
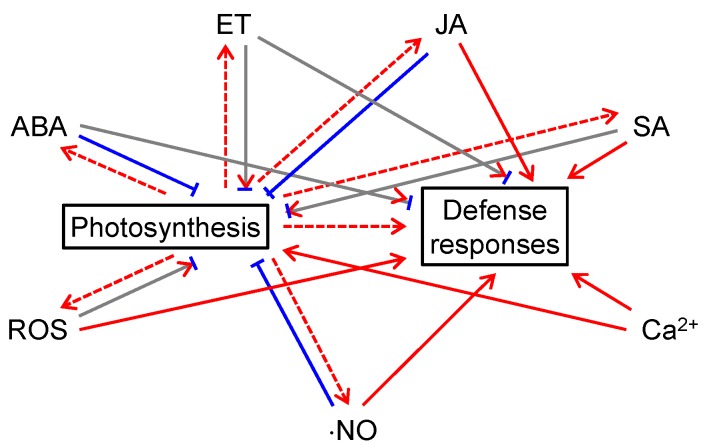
Interactions of photosynthesis and defense responses with defense–related signals. Red dotted arrows represent that photosynthesis provides electrons, NADPH, ATP, and/or carbon skeletons to the biosynthesis of defense hormones and other signals. Red arrow heads represent positive (promoting) effects; blue bars represent negative (inhibitory) effects; grey lines with red arrow heads and blue bars represent both positive and negative effects.

Extensive interactions exist between photosynthesis and defense-related signals (Figure 2). Photosynthetic electron transport supplies electrons to ROS producing enzymes; more importantly, photosynthesis provides NADPH, ATP, and carbon skeletons for the synthesis of defense-related compounds, including ABA, ET, JA, SA, and even ⋅NO (Figure 2). These defense-related hormones and signaling molecules may in turn influence photosynthesis (Figure 2). ABA, JA, and ⋅NO tend to have negative impacts on photosynthesis. Exogenous treatments with these molecules caused stomatal closure (or delayed stomatal opening) and reduced photosynthesis (or photosynthetic pigments) [61,62,63,137,249,250,292]. ET, SA, and ROS may have positive and negative impacts on photosynthesis. The effects of ET on photosynthesis are species-specific and age-dependent [110]. Similarly, the effects of SA on photosynthesis depend on plant species, treatment methods, treatment durations, and growth conditions [155]. The effects of ROS on photosynthesis are also multifaceted. On the one hand, ROS production by the photosynthetic electron transport chain has a protective role over photosynthetic complexes [15]. On the other hand, excess amounts of ROS can damage photosynthetic complexes, especially PSII, and thus result in photoinhibition [15,16,17,18,19,20,21,22,23,24,293]. Ca^2+^ generally has a positive impact on photosynthesis. The thylakoid membrane-localized CAS and stromal Ca^2+^ spikes are essential for the regulation and photoacclimation of photosynthesis [278].

## 6. The Participation of Different Plant Signals in Plant Defense against Pathogens

Phytohormones ABA, ET, JA, and SA, as well as ROS, ⋅NO, and Ca^2+^ directly or indirectly participate in plant defense against pathogens (Figure 2). ABA and ET may have positive and negative impacts on plant defense responses [6,82]. On the one hand, ABA induces stomatal closure and therefore blocks the entry of bacterial pathogens into plant tissues [28,29]. On the other hand, ABA has negative impacts on the post-invasion PTI response of plants [83,84]. Generally, ET inhibits the development of symptoms caused by necrotrophs and enhances the cell death caused by biotrophs and hemibiotrophs [125,126,127]. JA and SA have positive impacts on plant defense responses. JA is required in defense against necrotrophs and insect herbivores, and it acts synergistically with other hormones: JA and ET act synergistically against attacks by necrotrophs; JA and ABA act synergistically during herbivory [7,8]. SA mainly induces resistance to biotrophs and hemibiotrophs [10,11]. ROS, ⋅NO, and Ca^2+^ spikes generally play positive roles in plant defense against pathogens. The biphasic ROS accumulation is essential for plants to recognize pathogens, execute the hypersensitive response, and minimize the spread of pathogens [193]. ⋅NO potentiates ROS-induced localized cell death and induces the expression of defense-related genes [258]. Ca^2+^ spikes are involved in the development of both PTI and ETI responses [276].

## 7. PAMP Perception Induces Transcriptional Reprogramming of Nuclear-encoded Chloroplast-targeted Protein Genes

Plant pathogens elicit PAMPs, which are conserved among a particular class of microbes (e.g., flagella for bacterial pathogens and chitin for fungal pathogens) but are not produced by plants. The chemical nature of PAMPs could be polysaccharides, lipopolysaccharides, proteins, glycoproteins, and lipophilic substances [294]. Plants recognize these “non-self” molecules by pattern recognition receptors on the cell surface [295]. PAMP (e.g., flagellin and lipopolysaccharides) treatments were found to induce local and systemic SA accumulation and defense gene (e.g., *PR* genes) expression in *Arabidopsis* plants [296]. Further investigations showed that PAMP-induced SA accumulation requires functional ICS1, the key SA biosynthetic enzyme in the chloroplast, and that disruption of SA signaling significantly affected PAMP-triggered defense responses [297]. Researchers inoculated *Arabidopsis* leaves with *Pst* DC3000, T3SS-deficient mutant strains, and mock solution, and perform comparative transcriptome analysis at a series of time points (hours post inoculation) [33,35,36,297]. The transcriptomics revealed that PAMP recognition induces suppression of a relatively large number of nuclear-encoded chloroplast-targeted protein genes (e.g., photosynthesis-related genes) at early time points (i.e., two, three and four hours post infection) [35,36]. These data demonstrate that the chloroplast plays an early role in integrating pathogen and defense signals [35,36].

## 8. Chloroplasts are Targeted by Pathogen Effectors

Plant pathogens also elicit effector proteins to suppress host defense and promote pathogenicity [298,299]. For example, individual *P. syringae* strains use the T3SS to deliver approximately 15-30 T3Es into plants [300,301,302,303]. After entry into plant cells, some effectors move into discrete subcellular compartments, such as the plasma membrane, the endoplasmic reticulum, the nucleus, the tonoplast, vesicles, mitochondria, and chloroplasts [298,299,304,305]. A number of T3Es were found or proposed to localize to the chloroplast (Table 1) [35,305,306,307,308]. These T3Es act as virulence factors and manipulate chloroplast structure and functions. The N-terminal region of AvrRps4 (avirulence protein resistance to *P. syringae* 4) and HopK1 (Hrp outer protein K1) has been shown to be cleaved *in planta* and the processed AvrRps4 and HopK1 are localized in the chloroplast [308,309]. Although relevant experimental evidence is still lacking, HopO1-1, HopO1-2, and HopR1 (Hrp outer protein O1-1, O1-2, and R1) were predicted to have a cleavable transit peptide as well, according to LOCALIZER, a subcellular localization prediction program for plant proteins and pathogen effecters in plant cells [310]. HopI1 and HopN1 (Hrp outer protein I1 and N1) use a non-cleavable transit peptide to localize themselves to the chloroplast [306,307]. HopBB1 and HopM1 (Hrp outer protein BB1 and M1) are another two possibly chloroplast-targeted T3Es, although it is not yet clear whether they use a cleavable transit peptide (Table 1). To assist pathogen proliferation and virulence, these effectors manipulate chloroplast structure and functions, for example, remodel thylakoid membranes [306], reprogram the expression of nuclear-encoded chloroplast-targeted protein genes and chloroplast-encoded genes [35,36], disrupt photosynthetic water splitting, electron transport, and CO_2_ assimilation [35,307], minimize chloroplastic ROS production [37,307,308], alter enzyme redox status, and suppress SA accumulation [306].

### 8.1. AvrRps4 and HopK1

According to LOCALIZER, AvrRps4 and HopK1 contain an N-terminal cleavable chloroplast transit peptide [310]. However, confocal microscopic analysis of GFP-tagged effectors, chloroplast import assays of radiolabeled effectors, and subcellular fractionation of *Arabidopsis* plants expressing hemagglutinin-tagged effectors demonstrated that these two T3Es localize to multiple plant subcellular compartments: chloroplasts, the nucleus, and the cytoplasm [308,311]. The chloroplast fraction contains processed but not full-length forms of AvrRps4 and HopK1, the nuclear fraction contains full-length but not processed forms, and the cytoplasmic fraction mainly contains full-length forms [308]. While nuclear and cytoplasmic pools of AvrRps4 and HopK1 trigger immunity and the hypersensitive response, respectively [312], the chloroplastic pool is responsible for their virulence [308]. Consequently, *in planta* N-terminal processing of AvrRps4 and HopK1 and their chloroplast localization are required for their full virulence function, but are not required for their ability to induce immunity or the hypersensitive response [308]. Consistent with this hypothesis, expression of the AvrRps4 variant lacking the N-terminal transit peptide failed to localize the protein to the chloroplast; however, the protein retained its ability to induce the hypersensitive response [308]. Another piece of evidence is that the AvrRps4 processing-deficient mutant strain displayed reduced growth and milder disease symptoms, but still induced the hypersensitive response and immunity in plants [309].

AvrRps4 contains a KRVY motif (amino acids 135-138) [309] and a putative SSM4 E3 ubiquitin-protein ligase domain [331]. Mutations in the KRVY motif resulted in the abolishment of AvrRps4-triggered hypersensitive response and immunity, demonstrating that the KRVY motif is required for the avirulence activity of AvrRps4 [309]. Although the function of the SSM4 E3 ubiquitin ligase domain has not been experimentally demonstrated, many bacterial effectors manipulate the ubiquitination pathway [177,178,179,299]. Some act as deubiquitinases or E3 ligases and alter the function and stability of target proteins in the plant cell [177,178,179,299]; others regulate their own function, stability, and final destination [332,333,334,335].

AvrRps4 is detected by the R protein RPS4 (resistance to *P. syringae* 4) [313,316]. RPS4 may form complexes with EDS1 [312,313,314], a positive regulator of basal resistance and ETI. AvrRps4 targets EDS1 in the cytoplasm and the nucleus and disrupts the interactions of EDS1 with its partners such as RPS4 [312,313]. RPS4 may form complexes with another R protein, RRS1 (resistance to *Ralstonia solanacearum* 1) [316,336]. In the presence of RRS1, the RPS4-RRS1 complex associates the EDS1-PAD4 (phytoalexin deficient 4) or EDS1-SAG101 (senescence-associated gene 1) complex in the nucleus, and AvrRps4 does not disrupt their association [336]. In the absence of RRS1, AvrRps4 forms nucleocytoplasmic aggregates with EDS1, and the association between the two complexes is disrupted [336]. AvrRps4 also targets the WRKY domain of RPS1, activating RPS4-RRS1-dependent ETI, and targets the WRKY domain of transcription factors WRKY33, WRKY41, WRKY60, and WRKY70, interfering with the WRKY-dependent defense [317].

Taken together, AvrRps4 and HopK1 probably localize to multiple plant subcellular compartments, with the processed form localizing to the chloroplast and the full-length form localizing to the nucleus and the cytosol (e.g., cytoplasmic membranes). The chloroplast targets of AvrRps4 and the signature domain and *in planta* targets of HopK1 are not yet known. The requirement of the chloroplast localization and N-terminal processing for the ability of AvrRps4 and HopK1 to suppress PTI responses suggests that their virulence targets needed for immune suppression are located in the chloroplast [308]. As AvrRps4 and HopK1 suppress both early and late immune responses, components of chloroplast-to-nucleus retrograde signaling could be potential targets of AvrRps4 and HopK1 in the chloroplast [308].

### 8.2. HopO1-1, HopO1-2, and HopR1

According to LOCALIZER, HopO1-1, HopO1-2, and HopR1 also contain an N-terminal cleavable chloroplast transit peptide [310]. However, discrepancy exists in the experimental data. Confocal microscopic analysis of transiently expressed fluorescent protein-tagged effectors showed that HopO1s and HopR1 localize to the plasma membrane and the cytoplasm of plant cells, respectively [305,329]. However, de Torres Zabala et al. [35] demonstrated that in vitro translated HopO1-2 and HopR1 could be efficiently imported into pea chloroplasts. It is possible that HopO1-1, HopO1-2, and HopR1 localize to multiple plant subcellular compartments.

HopO1-1 and HopO1-2 contain an ART (Arg ADP-ribosyltransferase) domain [329]. *Pst* DC3000 mutant strains deficient in HopO1-1 or HopO1-2 displayed reduced *in planta* growth, indicating that these two T3Es are required for full virulence [329]. HopO1-1 and HopO1-2 variants with mutations in the ART domain were unable to suppress PTI and ETI, suggesting that the ART domain is essential for virulence [329]. Using a yeast-two-hybrid (Y2H) approach, HopO1-2 was found to interact with several non-chloroplastic plant proteins, including APC8 (anaphase-promoting complex subunit 8, an E3 ubiquitin ligase subunit), CSN5A (constitutive photomorphogenesis 9 signalosome 5A, a molecular regulator of E3 ubiquitin ligase), and OBE1 (oberon 1, a nuclear-targeted plant homeodomain finger protein) [318]. The biological significance of these interactions is not yet known. Furthermore, the *in planta* targets of HopO1-1 and the chloroplast targets of HopO1-2 are not yet identified.

HopR1 contains an AvrE (Avirulence protein E) effector domain and a SMC_N (N-terminus of structural maintenance of chromosomes proteins) domain [331]. Using the Y2H approach, HopR1 was found to interact with a number of nuclear-targeted proteins, such as JAZ3 (jasmonate-zim-domain protein 3), LSU1 (response to low sulfur 1), and TOE2 (target of early activation tagged 2) [35,318]. As a repressor in JA signaling, JAZ3 inhibits MYC2, a transcriptional activator of JA signaling [337]. However, JAZ3 appeared to be a positive regulator of plant defense, as the *Arabidopsis jaz3* mutant displayed enhanced susceptibility to *P. syringae* and the oomycete *Hyaloperonospora arabidopsidis* [318]. Loss-of-function *Arabidopsis* mutants of LSU2, a homolog of LSU1, also displayed enhanced susceptibility to *P. syringae* and *H. arabidopsidis* [318]. TOE2 is a transcription factor capable of interacting with a subset of JAZ repressors (JAZ1/3/4/9) [338]. These observations suggest that HopR1 may manipulate plant nuclear gene expression to assist pathogenicity.

HopR1 also interacted with the chloroplastic protein PTF1 in the Y2H assay [35,318]. PTF1 is able to bind to the blue light-responsive promoter region of the *psbD* gene [67], which encodes the D2 protein of PSII. In the *Arabidopsis ptf1* mutant, the accumulation of the *psbD* transcript is significantly reduced; consequently, the *ptf1* mutant displayed pale green cotyledons and retarded growth [67]. Because of the essential role of PTF1 in regulating photosynthesis, it is tempting to hypothesize that HopR1 modulates photosynthesis via interaction with PTF1. Another chloroplastic protein that interacted with HopR1 in the Y2H assay was CBSX2, a cystathionine β-synthase domain-containing protein [35,318]. CBSX2 is highly homologous to CBSX1, a protein involved in the activation of plastidial thioredoxins [339]. Plastidial thioredoxins are thiol-based redox regulators of many chloroplastic processes, including chlorophyll biosynthesis, light and carbon fixation reactions of photosynthesis, and H_2_O_2_ scavenging [339,340]. Therefore, it is possible that HopR1 may localize to the chloroplast, interact with chloroplastic proteins such as PTF1 and CBSX2, and manipulate chloroplastic processes to assist pathogenicity.

### 8.3. HopI1 and HopN1

HopI1 and HopN1 are chloroplast-targeted *P. syringae* T3Es that enter the chloroplast via a noncanonical mechanism and are not processed after entry [306,307,320,321,328]. Confocal microscopic analysis of transiently expressed fluorescent protein-tagged effectors showed that HopI1 and HopN1 localize to the chloroplast [306,307]. The chloroplast localization of these two T3Es was further confirmed with subcellular fractionation [306,307]. It should be noted that transiently expressed HopN1-YFP protein was previously found in the plasma membrane of Chinese cabbage and tobacco epidermal cells [341]. One possible reason for the discrepancy is the absence of chloroplasts in epidermal cells [307].

HopI1 is a DnaJ protein with a phosphate-binding loop (P-loop), a proline and glutamine (PQ)-rich repeat region, and a J-domain with a histidine-proline-aspartate (HPD) motif [306,320]. HopI1 was found to cause thylakoid membrane remodeling and suppression of SA accumulation and SA-dependent defenses [306]. As J-domains are known to interact with 70 kDa heat shock proteins (Hsp70s) and activate their ATPase activity and protein folding ability [342], Hsp70s were proposed to be the target of HopI1 [306]. Consistent with this hypothesis, the J-domain of HopI1 from *P. syringae* pv. *maculicola* strain ES4326 was found to bind to full-length plant cytHsp70-1 (cyt stands for cytosolic) in vitro, and full-length HopI1 successfully pulled down cpHSP70-1 (cp stands for chloroplast) from pea chloroplasts [321]. In addition, the J-domain of HopI1 stimulated the ATP hydrolysis activity of Hsp70s [321]. Furthermore, immunoprecipitation showed that HopI1 interacts with both cytHsp70-1 and cpHsp70-1, recruits cytHsp70-1 to the chloroplast, and forms large complexes with cytHsp70-1 and cpHsp70-1 [321]. The involvement of cytHsp70-1 in this process is interesting. It is possible that the level of cpHsp70-1 is not high enough for HopI1 to function without recruiting cytHsp70-1 to the chloroplast [321]. Indeed, genetics studies showed that cytHsp70-1 is required for the virulence role of HopI1 at standard growth temperatures [321]. Therefore, it was hypothesized that, in the absence of HopI1, cytHsp70-1 and cpHsp70-1 may act in basal defense by facilitating folding and complex assembly of chloroplast-targeted defense factors, such as components of SA biosynthesis and transport [321]. Upon pathogen infection, HopI1 may switch these Hsp70s to function in degradation or disassembly of defense-promoting complexes [321].

HopN1 is a Cys protease with experimentally confirmed proteolytic activity [328]. This effector was found to suppress PCD, ROS production, and callose deposition in plants [307,328]. The *Pst* DC3000 *hopN1* mutant strain with altered Cys protease catalytic triad lost its ability to suppress the hypersensitive response, indicating that the Cys protease domain is essential for hopN1 to suppress plant cell death [328]. In vitro pull-down assays identified PsbQ (protein Q in photosystem II oxygen evolution complex) as the target of HopN1 [307]. HopN1 demonstrated proteolytic activity towards PsbQ in *N. benthamiana* thylakoids [307]. In addition, chloroplasts isolated from HopN1-expressing tomato leaves displayed reduced PSII activity [307]. In line with these observations, PsbQ-silenced tobacco plants showed reductions in bacterium-induced ROS production and cell death [307]. Therefore, HopN1 may reduce photosynthetic water splitting, oxygen production, electron transport, and ROS generation in the chloroplast by degrading PsbQ [307].

### 8.4. HopBB1 and HopM1

Another two possibly chloroplast-targeted T3Es are HopBB1 and HopM1 (Table 1). Although the subcellular location of HopBB1 in the plant cell has not been experimentally investigated, HopBB1 was found to interact with some nuclear and chloroplastic proteins [35,318]. Therefore, HopBB1 may localize to multiple plant subcellular compartments. Discrepancy exists in experimental data on the localization of HopM1 in the plant cell. Using confocal microscopic analysis of fluorescent protein-tagged effector, Nomura et al. [324] showed that HopM1 localizes to the trans-Golgi network/early endosome compartment. However, Choi et al. [305] showed that HopM1 localizes to the chloroplast, with a similar approach. The cause of this discrepancy is not clear. It is possible that HopM1 localizes to multiple plant subcellular compartments and different localizations trigger different responses.

HopBB1 is a T3E with a putative AvrPphF-ORF2 domain (AvrPphF is a homolog of HopF2 in *P. syringae* pv. *phaseolicola*; ORF stands for open reading frame) [331,343]. The AvrPphF-ORF2 domain is structurally homologous to the catalytic domain of bacterial ADP-ribosyltransferases [343], although purified AvrPphF-ORF2 did not show ADP-ribosyltransferase activity [344]. Using the Y2H approach, HopBB1 was found to interact with a number of nuclear proteins, including JAZ3, TCP14 and TCP15 (teosinte branched/cycloidea/PCF 14 and 15) [35]. Similar to other JAZ repressors, JAZ3 is a direct target of the SCF^COI1^ E3 ubiquitin ligase during JA signaling [337]. TCP14 is a repressor for the transcription of a subset of JA response genes [319]. The interactions of HopBB1 with JAZ3 and TCP14 were confirmed with multiple independent approaches [319,345]. More importantly, HopBB1 was found to “glue” together JAZ3 and TCP14, two repressors of JA signaling, and target them for degradation by the SCF^COI1^-dependent ubiquitination [319]. HopBB1 was also found to interact with PTF1 in the Y2H assay [318]. Because of the essential role of PTF1 in regulating photosynthesis, it is tempting to hypothesize that HopBB1 modulates photosynthesis via interaction with PTF1. Further studies are needed to investigate the biological significance of the HopBB1-PTF1 interaction.

HopM1 was found to interact with MIN7 (HopM1 interactor 7), an ADP-ribosylation factor-guanine nucleotide exchange factor, in the Y2H assay [325]. MIN7 is a key controller of vesicle trafficking and it is involved in PTI, ETI, and SA-mediated immunity [324,325]. The interaction between HopM1 and MIN7 was confirmed by the pull-down assay [325]. HopM1 targets MIN7 via the N terminus, promotes ubiquitination and destruction of MIN7 via host cell 26S proteasomes, and thus suppresses vesicular trafficking during plant defense [325]. In line with these results, MIN7 was found to co-localize with HopM1 to the trans-Golgi network/early endosome compartment of plant cells and ETI was found to block the degradation of MIN7 in resistant plants [324]. HopM1 also interacted with MIN10, a 14-3-3 protein, in the Y2H assay [325]. MIN10 and other 14-3-3 proteins are required for early PTI responses, namely, stomatal immunity and PAMP-triggered ROS production [326]. HopM1 preferably eliminated endomembrane-associated MIN10, suggesting that HopM1 may target and destabilize MIN10 in plant endomembranes [325]. In agreement with this hypothesis, HopM1 was found to suppress early PTI responses and this effect is independent of MIN7 [326]. Therefore, HopM1 may suppress early PTI responses in a MIN7-independent manner, by destabilizing MIN10 and other 14-3-3 proteins [326]. HopM1 was also proposed to target and promote the destruction of *Arabidopsis* response regulator 2 (ARR2) [327], a transcription factor capable of binding to TGA3 (TGA1a-related gene 3) and activating *PR* gene expression [346,347]. Consistent with this hypothesis, HopM1 was found to suppress the expression of *PR* genes and this HopM1-mediated suppression was absent in the loss-of-function mutant of TGA3 [327]. Taken together, two confirmed and one potential targets have been identified for HopM1, although the chloroplast target(s) of HopM1 have not been discovered yet.

## 9. Chloroplast Structure and Functions are Manipulated by Phytotoxins

Plant pathogens also produce phytotoxins to suppress host defense and promote pathogenicity [30]. Among the five most extensively investigated *P. syringae* phytotoxins, syringomycin and syringopeptin form ion channels in the plant cell plasma membrane, causing cytolysis and necrosis [30]. Although the subcellular distribution of tabtoxin, phaseolotoxin, and coronatine has not been experimentally investigated, these three phytotoxins were found to affect chloroplast structure and functions and cause chlorosis [30]. Tabtoxin inhibits cytosolic and chloroplastic glutamine (Gln) synthetase [348,349]. The chloroplastic Gln synthetase re-assimilates photorespiratory ammonia. Free ammonia dissipates pH gradients across biological membranes, causing disruption of thylakoid membranes, uncoupling of photophosphorylation, and ultimately, chlorosis of plant tissues [350]. Phaseolotoxin inhibits ornithine carbamoyltransferase [351,352,353,354], which converts ornithine and carbamoyl phosphate to citrulline in the chloroplast [355]. Inhibition of ornithine carbamoyltransferase results in over-accumulation of ornithine, deficiency of Arg, blockage of translation, reduced chlorophyll synthesis, and chlorosis of plant tissues [353,354]. Consequently, photosynthesis is limited by the very low level of chlorophyll at the site of infection [354].

COR activates JA signaling by mimicking JA-Ile, the endogenous bioactive jasmonate [356]. Like JA-Ile, COR is capable of binding to the JA co-receptor complex, which contains the JA receptor COI1 and a JAZ repressor [357]. The binding triggers SCF^COI1^-mediated ubiquitination and 26S proteosome-mediated degradation of JAZ repressors [42,337,358]. Upon destruction of JAZs, positive transcription regulators, such as MYC2/3/4 and MYBs, are relieved from repression and activate the expression of JA-responsive genes [8]. One example of MYC-regulated genes is NAC (NAM, ATAF, and CUC) transcription factors (e.g., ANAC019/055/072), which are involved in COR-induced stomatal reopening and chlorophyll degradation [359,360]. NAC transcription factors also suppress the accumulation of SA by repressing the SA biosynthetic gene *ICS1* and activating the SA modifying gene *BSMT1* (*benzoic/salicylic acid carboxyl methyltransferase 1*) [359]. Chlorophyll catabolic genes (e.g., *SGR1* [*stay-green 1*]) are also regulated by MYCs [360,361]. COR suppressed plant defense by disabling stomatal defense [28], inducing *SGR1* expression and causing chlorophyll degradation [361], and inhibiting SA accumulation [359], in COI1-dependent manners. COR treatment also caused leaf growth arrestment, repression of photosynthetic genes, and a transient reduction of PSII quantum yield at the following dawn, presumably due to delayed stomatal opening at the night-day transition [292]. Interestingly, COR was found to suppress callose deposition, enhance bacterial growth, and promote bacterial virulence in the COI-deficient mutant, suggesting that COR has other plant target(s) besides the COI1-JAZ complex [362]. The subcellular distribution of COR in the plant cell has been experimentally investigated. Because COR binds to the COI1-JAZ complex, it is conceivable that at least a fraction of COR localizes to the nucleus. Using electron microscopy and immunogold labeling, COR was also found to be associated with chloroplasts in plants infected by *P. syringae* [363]. Therefore, it was hypothesized that COR may translocate to the chloroplast and interact with chloroplast-associated proteins during JA/COR signaling [364].

## 10. Summary: The Chloroplast Plays a Central Role in the Interplay between Photosynthesis, Pathogen infection, and Plant Defense

As the site of photosynthetic light reactions and carbon fixation reactions, the chloroplast is indispensible for photosynthesis. The chloroplast is also a major generator of defense-related signaling molecules or their precursors [365]. Early steps of ABA [4,5], ET [6], and JA [7,8] biosynthesis occur in the chloroplast; SA is primarily synthesized in the chloroplast [11]; ROS could be produced by PSI and PSII [15,16]; ⋅NO could be made by the nitrate reductase and ⋅NO synthase-like enzyme in the chloroplast [234,235]; and Ca^2+^ spikes could be generated by thylakoid-membrane-localized CAS [276]. The fact that photosynthesis and biosynthesis of these defense-related signaling molecules occur in the same organelle facilitates the interactions between photosynthesis and defense signaling. On the one hand, synthesis of these defense-related signaling molecules requires photosynthetic products such as carbon skeletons, energy, and reducing power, which are conveniently available in the chloroplast. On the other hand, these defense-related signals may influence photosynthesis by regulating the expression of chloroplast-encoded photosynthetic genes and nuclear-encoded chloroplast-targeted photosynthetic protein genes.

Chloroplasts are targeted by pathogen effectors [35,308,366]. In order to translocate into chloroplasts, some effectors (e.g., ArvRps4 and HopK1 [308,309]) utilize an N-terminal cleavable chloroplast transit peptide, and some other effectors (e.g., HopI1 and HopN1 [306,307]) employ a non-cleavable transit peptide. Most chloroplast-targeted effectors also translocate to other subcellular compartments of plant cells. For effectors with a cleavable chloroplast transit peptide, their chloroplast localization and *in planta* N-terminal processing are essential for their full virulence [308,309]. After entry into chloroplasts, these effectors may interact with chloroplastic proteins (e.g., PTF1, CBSX2, Hsp70, and PsbQ) and manipulate chloroplast structure and functions (e.g., thylakoid remodeling, expression of photosynthetic genes, photosynthetic water splitting and electron transport, enzyme redox status, and SA biosynthesis).

Pathogens also produce phytotoxins to inhibit host defense and enhance pathogenicity; some phytotoxins affect the structure and functions of chloroplasts. Tabtoxin [348,349] and phaseolotoxin [351,352,353,354] inhibit the activities of two chloroplastic enzymes, Gln synthetase and ornithine carbamoyltransferase, respectively. COR, on the other hand, modulates plant JA signaling, promotes chlorophyll degradation, and inhibits SA biosynthesis in the chloroplast [359,360,367]. These three phytotoxins ultimately cause chlorosis of plant tissues [30]. Taken together, these studies suggest that the chloroplast plays a pivotal role in the interplay among photosynthesis, pathogen infection, and plant defense.

## 11. Future Perspectives and Outstanding Questions

To sum up, chloroplasts play an early and important role in integrating pathogen and defense signals, regulating the interplay among pathogen infection, plant defense, photosynthesis, and other biological processes. Protecting chloroplasts from pathogen effectors and phytotoxins could be a strategy to broad-spectrum resistance to plant pathogens [30,35]. However, there are still several unresolved questions. (1) How chloroplasts integrate intracellular (e.g., defense-related signaling molecules) and external signals (e.g., pathogen effectors and phytotoxins) and communicate with other organelles, to attain synchronized whole-cell defense responses upon pathogen infection, is not entirely clear. (2) The subcellular location of some putatively chloroplast-targeted effectors (e.g., HopBB1) in the plant cell still requires experimental verification. (3) The chloroplast or *in planta* targets of some effectors, such as AvrRps4, HopK1, HopO1-1, HopO1-2, and HopM1, have not been identified yet. (4) The Y2H screening approach identified a number of potential protein targets for the nine effectors listed in Table 1. Some of these interactions (e.g., HopR1 with PTF1 and CBSX2, HopBB1 with PTF1) await verification with different experimental approaches. Additionally, the biological significance of these interactions needs to be explored further. (5) Although tabtoxin and phaseolotoxin have been shown to inhibit the activities of two chloroplastic enzymes, their subcellular distribution in the plant cell has not been investigated yet.

## Figures and Tables

**Table 1:** Known and potential plastid-targeted bacterial effectors.

Name	Full Length (aa)	Cleavable Transit Peptide?	Signature Domain	Known and Potential Target Proteins	Function	References
AvrRps4	221^a^	Yes, 133 aa	SSM4	EDS1	Disrupts the interactions of EDS1 with its partners, such as RPS4	[308,309,311,312,313,314,315]
				WRKY domain of RRS1, WRKY33, WRKY41, WRKY60, WRKY70	Activates RPS4-RRS1-dependent ETI; interferes with WRKY-dependent defense	[316,317,318]
HopBB1	280^b^	Not sure	AvrPphF-ORF2	JAZ3, TCP14	Promotes TCP14 and JAZ3 degradation; de-represses TCP14-regulated JA response	[35,318,319]
				PTF1^d^	Modulates *psbD* expression?	[318]
				TCP15^d^, TOE2^d^, UNE12^d^	Not known yet	[318]
HopI1	488^a^	Non-cleavable transit peptide	DnaJ	cytHsp70-1, cpHsp70-1	Recruits cytHsp70-1 to chloroplasts, forms complexes with cytHsp70-1 and cpHsp70-1, and activates their ATPase activity	[306,320,321]
HopK1	338^a^	Yes, 133 aa	Not identified yet	Not identified yet	Not known yet	[308,322,323]
HopM1	712^a^	Not sure	Not identified yet	MIN7, MIN10	Degrades MIN7 and MIN10	[305,324,325,326]
				ARR2?	Degrades ARR2 and suppresses *PR* gene expression?	[327]
				At3g11720^d^	Not known yet	[318]
HopN1	350^a^	Non-cleavable transit peptide	Cys protease	PsbQ	Degrades PsbQ	[307,328]
HopO1-1	283 ^a^	Yes, 28–72 aa^c^	ART	Not identified yet	Not known yet	[310,323,329]
HopO1-2	298^a^	Yes, 40–87 aa^c^	ART	APC8^d^, CSN5A, OBE1^d^, At5g16940^d^	Not known yet	[35,310,318,323,329]
HopR1	1957^a^	Yes, 52–79 aa^c^	AvrE, SMC_N	LSU1^d^, JAZ3^d^, TOE2^d^	Manipulates nuclear gene expression?	[35,305,310,318,330]
				PTF1^d^	Modulates *psbD* expression?	[318]
				CBSX2^d^	Regulates redox status of chloroplastic enzymes?	[318]
				DUT1^d^, LSU3^d^, At3g48550^d^, At4g17680^d^	Not known yet	[318]

^a^ The protein sequence is from *S. syringae* pv. *tomato*; ^b^ The protein sequence is from *S. syringae* pv. *spinaceae*; ^c^ The length of the chloroplast transit peptide was predicted by LOCALIZER [310]; ^d^ Potential target proteins identified via Y2H assays.
